# A Complex Case of Glucose-Phosphate Isomerase Deficiency With an Indeterminate Adrenal Incidentaloma

**DOI:** 10.7759/cureus.104615

**Published:** 2026-03-03

**Authors:** Gauri Satheesh Nair, Nisha R Sungar, Naveen Rojed, Azhar Rizvi, Balasubramanian Thiagarajan Srinivasan

**Affiliations:** 1 Medicine, United Lincolnshire Teaching Hospitals NHS Trust, Lincoln, GBR; 2 Acute Medicine, Lincoln County Hospital, Lincoln, GBR; 3 Radiology, United Lincolnshire Teaching Hospitals NHS Trust, Lincoln, GBR; 4 Endocrinology and Diabetes, Lincoln County Hospital, Lincoln, GBR; 5 Endocrinology and Diabetes, United Lincolnshire Teaching Hospitals NHS Trust, Lincoln, GBR

**Keywords:** adrenal incidentaloma investigations, extra-medullary hematopoiesis, gpi deficiency, multi-disciplinary teams, pet ct scan

## Abstract

Adrenal incidentalomas are being identified more frequently with the widespread use of cross-sectional imaging, and their assessment requires careful evaluation to exclude hormonal excess and malignancy. We present the case of a middle-aged woman with chronic hemolytic anaemia due to glucose-phosphate isomerase (GPI) deficiency who was found to have a gradually enlarging left adrenal mass during long-term follow-up. Over an 11-year period, the lesion increased in size from 1.2 cm to 6 cm, appropriately raising concern about malignant potential. The presence of additional paravertebral soft-tissue lesions added further diagnostic uncertainty. Through a multidisciplinary approach involving endocrinology, haematology, specialist radiology, and regional multidisciplinary team discussion, supported by detailed biochemical assessment and positron emission tomography-computed tomography (PET-CT) imaging, the lesion was ultimately characterised as a biochemically non-functioning adrenal incidentaloma with low metabolic activity. This case underscores the value of multidisciplinary collaboration and highlights the importance of recognising extramedullary hematopoiesis as a potential radiological mimic of malignancy in patients with chronic hemolytic disorders.

## Introduction

Adrenal incidentalomas are detected in up to 4% of abdominal CT scans and require structured hormonal and radiological evaluation to stratify malignancy risk and functional status [1]. Larger lesions (>4 cm), interval growth, heterogeneous morphology, or atypical imaging characteristics warrant further assessments. Modern endocrine guidelines emphasise structured biochemical testing - to assess cortisol, catecholamine, and aldosterone secretion; and specialist radiological evaluation - to determine whether surgery, surveillance, or multidisciplinary team (MDT) review is required as appropriate [2-4].

Glucose-phosphate isomerase (GPI) deficiency is a rare autosomal recessive glycolytic enzymopathy causing chronic non-spherocytic hemolytic anaemia [5-7]. Chronic hemolysis and transfusion dependence can lead to iron overload and predispose to extramedullary hematopoiesis (EMH), often occurring in paravertebral regions [8]. EMH may mimic metastatic disease radiologically and complicate the evaluation of adrenal masses [9-11].

Diagnostic interpretation is further influenced by evolving adrenal incidentaloma guidelines; criteria for radiological risk stratification, recommended biochemical screening, and thresholds for intervention have changed significantly over the last decade [2-4,11,12]. As a result, adrenal nodules that were previously dismissed may now meet criteria for full endocrine evaluation.

This case highlights these complexities by presenting an enlarging, heterogeneous adrenal incidentaloma in a patient with GPI deficiency, illustrating how updated guideline-based assessment, longitudinal biochemical evaluation, and MDT collaboration can refine diagnostic accuracy and support safe, conservative management.

## Case presentation

The patient is a woman in her mid-40s with a known history of GPI deficiency, chronic hemolytic anaemia, prior splenectomy, transfusion dependence, and iron overload. GPI deficiency is a rare autosomal recessive enzymopathy associated with chronic non-spherocytic hemolysis [5-7]. She remained under routine haematology follow-up and had no clinical symptoms suggestive of catecholamine excess (e.g., palpitations, diaphoresis), cortisol overproduction, or mineralocorticoid excess.

**Figure 1:**
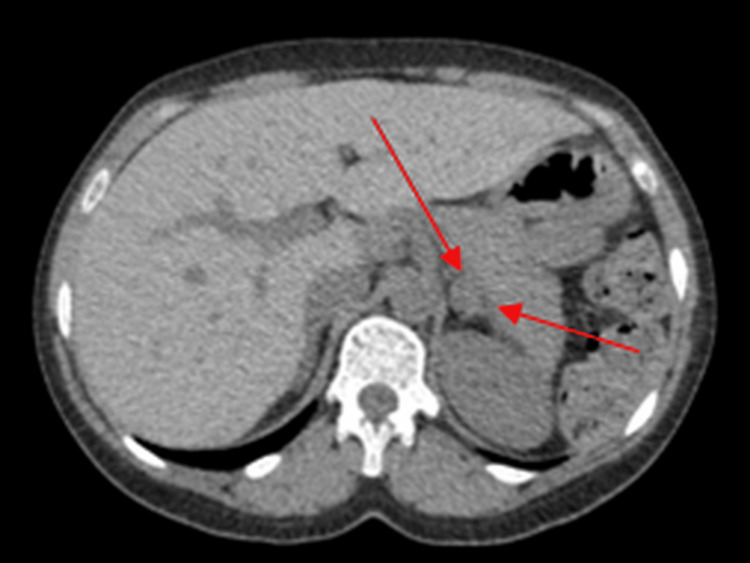
Initial computed tomography (CT) of the adrenal gland demonstrating a 1.2-cm adrenal mass (red arrows).

In 2017, during imaging performed for unrelated abdominal symptoms, a 1.2 cm left adrenal nodule was visualised on CT (Figure 1). The lesion was homogeneous and non-specific, with no radiological features mandating endocrine referral based on adrenal incidentaloma guidelines [1-4]. No further endocrine evaluation was pursued, and the lesion was collectively documented as an incidental, clinically insignificant finding.

In mid-2025, the patient underwent a cardiac MRI as part of routine assessment for iron overload. This scan again visualised the left adrenal lesion, which was now substantially larger than in 2017. The MRI also suggested heterogeneous internal characteristics, raising suspicion for an atypical adrenal adenoma, a potentially malignant adrenal neoplasm or another non-adenomatous adrenal pathology.

Given these changes, the patient was re-referred to endocrinology for formal assessment, consistent with established criteria for re-evaluation of adrenal masses [2-4].

Imaging evaluation

**Figure 2:**
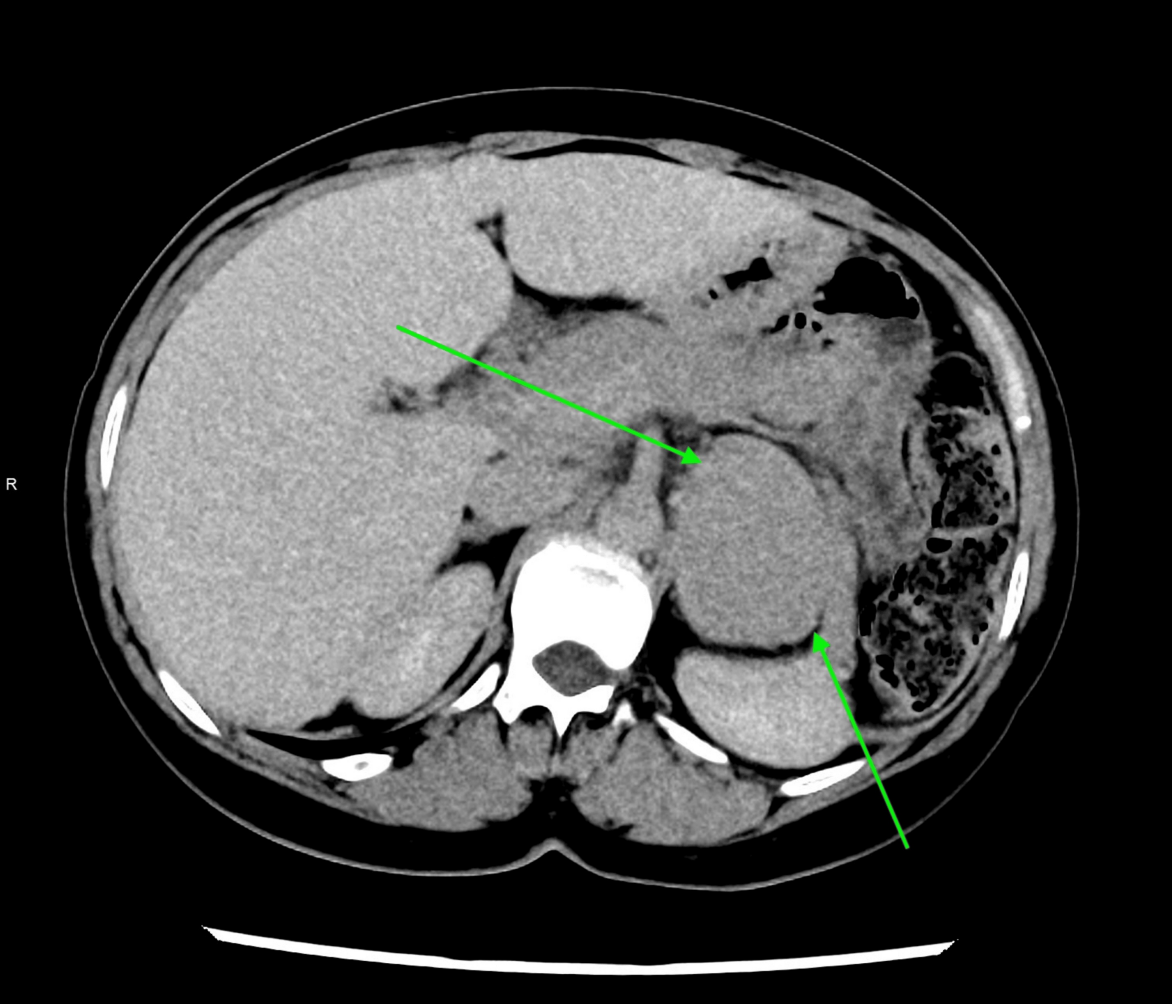
Interval increase in the size of the adrenal mass on follow-up imaging (2025) (green arrows).

During subsequent follow-up, the adrenal lesion was re-identified on imaging and demonstrated progressive enlargement. By July 2025, CT adrenal protocol imaging showed a 6.0 × 4.3 × 5.3 cm mixed solid-cystic left adrenal mass with attenuation values and contrast washout characteristics not typical of a lipid-rich adenoma (as shown in Figure 2). Given the lesion size, interval growth, and indeterminate imaging features, concern for malignant potential was raised.

**Figure 3:**
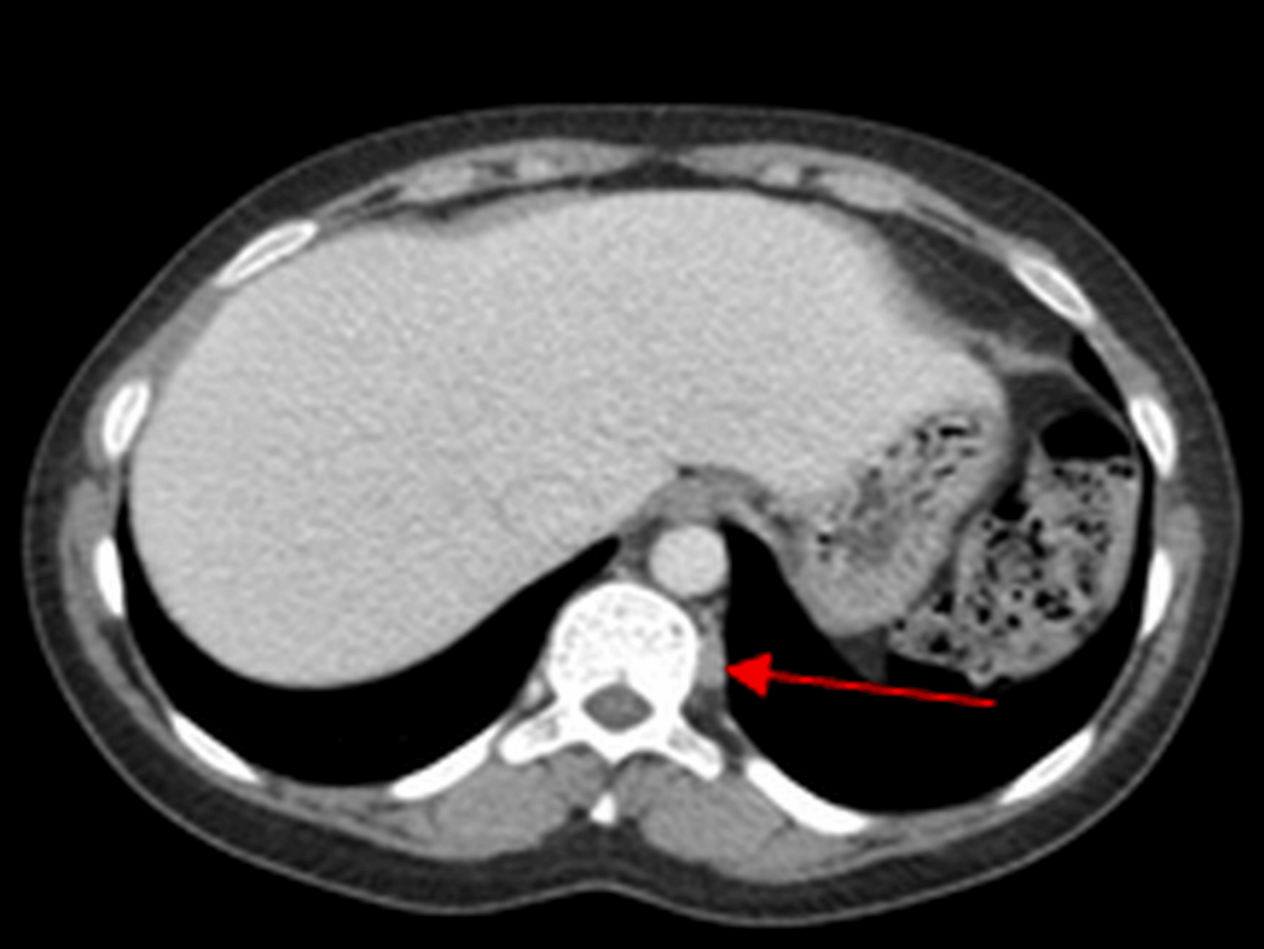
Computed tomography (CT) image from 2013 demonstrating the paravertebral lesion (arrow).

**Figure 4:**
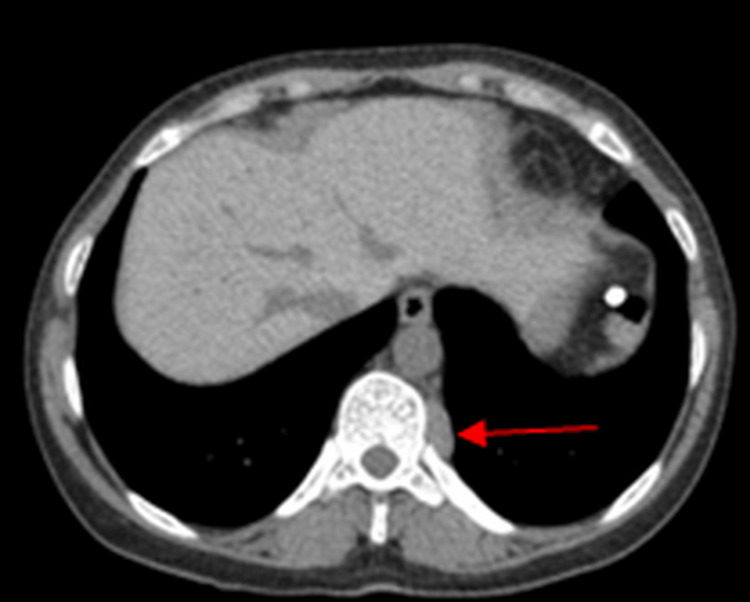
Computed tomography (CT) image from 2017 demonstrating an increase in the size of the paravertebral lesion (arrow).

Additionally, two left paravertebral soft-tissue masses at the T9-T10 levels were identified on surveillance CT scans, demonstrating progression over time. These lesions were first noted on a CT scan obtained in 2013 (Figure 3) and showed progression on a subsequent CT scan performed in 2017 (Figure 4).

This prompted differential diagnoses, including metastatic disease or neurogenic tumour.

**Figure 5:**
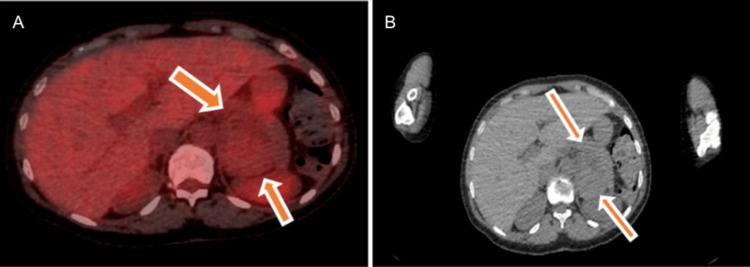
(A, B) Positron emission tomography-computed tomography (PET-CT) and CT images demonstrating the adrenal lesion with minimal fluorodeoxyglucose (FDG) uptake (arrows).

**Figure 6:**
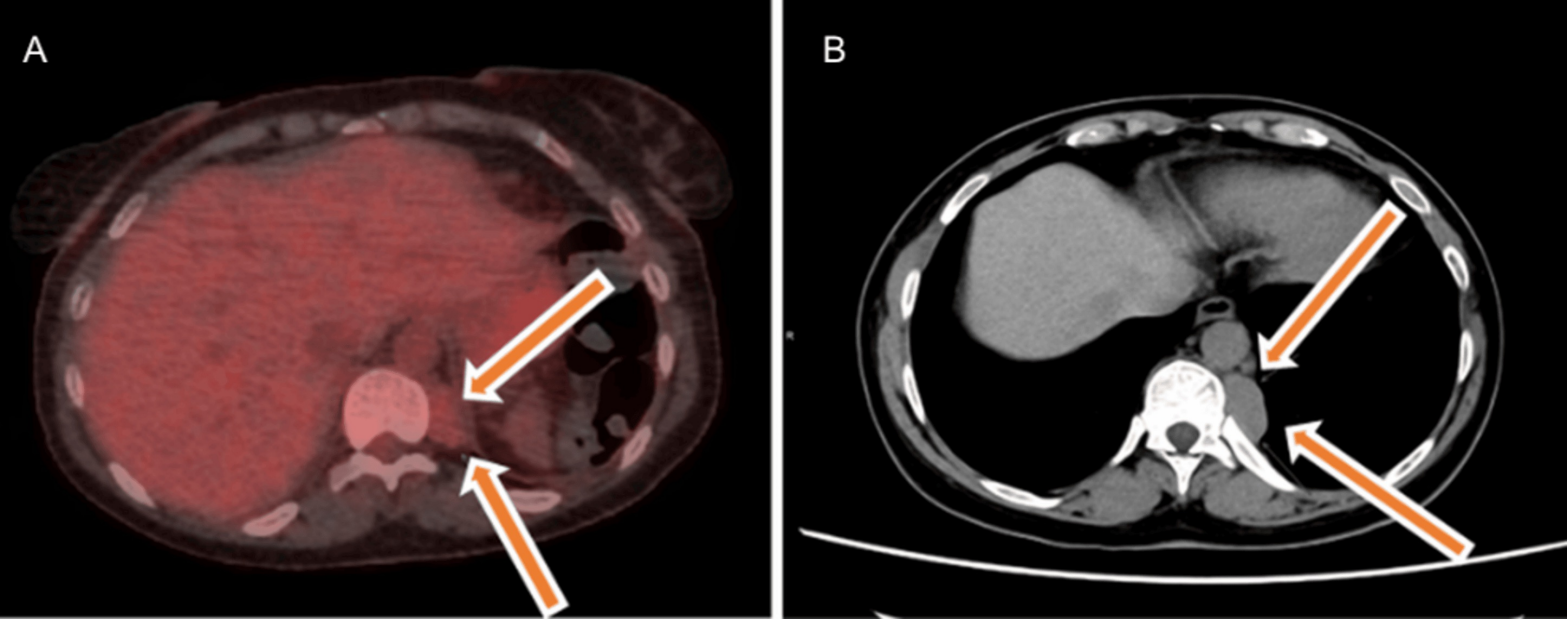
(A, B) Positron emission tomography-computed tomography (PET-CT) and CT images demonstrating the paravertebral lesions (arrows).

A whole-body positron emission tomography-computed tomography (PET-CT) was undertaken as part of the MDT discussion. This demonstrated low-grade fluorodeoxyglucose uptake within the adrenal lesion (SUVmax 2.5) (as shown in Figure 5) and within the paravertebral lesions (SUVmax 2.9), values below those typically associated with adrenal malignancy (as shown in Figure 6) [12]. Following specialist radiology review, the paravertebral lesions were considered most consistent with EMH.

Endocrine evaluation

Comprehensive biochemical evaluation was performed, including repeated 24-hour urinary metanephrines, normetanephrines, and 3-methoxytyramine, all of which were within reference ranges. Screening for cortisol excess and assessment of the aldosterone-renin ratio were done, excluding hormonally active adrenal disease or Conn's syndrome (Table 1).

**Table 1:** Biochemical evaluation for the suspected functional adrenal mass. DHEA-S: dehydroepiandrosterone sulfate

Test	Result	Reference Range	Interpretation
Overnight dexamethasone suppression test (DST) - cortisol	<50 nmol/L	<50 nmol/L	Adequate suppression; no evidence of autonomous cortisol secretion
24-hour urinary metadrenaline	0.82	0-1.29 umol/24hour	No biochemical evidence of pheochromocytoma
24-hour urinary normetadrenaline	2.01	0-3.69 umol/24 hour	No biochemical evidence of pheochromocytoma
24-hour urinary 3-methoxytyramine	1.05	0-2.59 umol/24hour	No elevation suggestive of a dopamine-secreting tumour
Plasma aldosterone	166 pmol/lt	Lab-specific	-
Plasma renin	1.1 nmol/lt/hr	Lab-specific	-
Aldosterone-renin ratio (ARR)	151	ARR < laboratory cut-off	No evidence of primary aldosteronism
Serum potassium	4.4	3.5-5.0 mmol/L	Supports the absence of hyperaldosteronism
HbA1c	Normal	<42 mmol/mol	No biochemical hypercortisolism-related dysglycaemia
DHEA-S	Normal	Age-adjusted reference interval	No features suggestive of adrenocortical carcinoma

Taken together, the biochemical and imaging findings supported the diagnosis of a biochemically non-functioning adrenal incidentaloma with low malignant potential.

## Discussion

Diagnostic evaluation of adrenal incidentalomas has evolved considerably over the past decade, contributing to the complexity of interpreting this patient’s enlarging lesion. Earlier clinical practice relied heavily on size thresholds - traditionally >4 cm - as the main indicator of malignancy risk, often prompting surgical consideration even when biochemical testing was unremarkable [11]. More recent guidance, such as the 2023 European Society of Endocrinology (ESE) guidelines, now emphasises a more nuanced approach that integrates lesion morphology, non-contrast Hounsfield units, contrast washout characteristics, interval growth, and MDT review rather than size alone. Radiology-focused recommendations have also highlighted how heterogeneous lesions, irregular borders, or atypical enhancement patterns should prompt further evaluation beyond size cut-offs [3,12].

These evolving criteria help explain why the small adrenal lesion visualised incidentally in 2017 did not trigger extensive biochemical or radiological assessment at that time. Earlier diagnostic frameworks would not have mandated full endocrine testing or MDT review for a small, non-lipid-rich lesion without suspicious clinical features [1,3]. As modern evaluation strategies increasingly prioritise structured hormonal testing and radiological characterisation [2-4], the patient’s lesion appropriately underwent detailed assessment in 2025 once it demonstrated significant interval growth and heterogeneous morphology - features that now prompt concern for malignancy under updated algorithms.

The coexistence of paravertebral lesions created additional diagnostic uncertainty. EMH can closely mimic metastatic disease radiologically, particularly in patients with chronic hemolytic disorders [8-10]. Older guidance sometimes supported biopsy of adrenal lesions when metastasis was suspected; however, modern recommendations caution against biopsy unless metastatic disease is strongly suspected and pheochromocytoma has been biochemically excluded [4]. The decision to use PET-CT, repeat biochemical profiling, and specialist radiology review aligns with contemporary best practice. This case demonstrates how evolving guidelines and MDT-based decision-making play a pivotal role in interpreting adrenal incidentalomas, particularly when the clinical context is complex.

## Conclusions

This case highlights the structured, stepwise diagnostic approach required when evaluating a complex adrenal incidentaloma, particularly in patients with underlying hematologic disorders such as GPI deficiency. Although the combination of chronic hemolysis, EMH, and an enlarging heterogeneous adrenal mass is exceptionally rare, the principles guiding evaluation are broadly applicable. These include reviewing longitudinal imaging for interval change, applying modern adrenal incidentaloma guidelines, conducting targeted biochemical testing for cortisol, catecholamine, and aldosterone excess, and interpreting findings through updated radiological criteria such as Hounsfield units, washout characteristics, and PET-CT metabolic activity. MDT collaboration - integrating endocrinology, radiology, and haematology - was essential in synthesising these results, distinguishing benign processes such as EMH from malignancy, and avoiding unnecessary surgical intervention. This case therefore reinforces the importance of systematic hormonal assessment, contemporary imaging interpretation, and multidisciplinary decision-making as key components in the safe and effective management of adrenal incidentalomas in both typical and atypical clinical contexts.
